# Apolipoprotein A‐I (CSL112) and Cardiovascular Outcomes in Atherosclerotic Cardiovascular Disease: A Scoping Review

**DOI:** 10.1002/clc.70305

**Published:** 2026-04-28

**Authors:** Awais Habib, Muhammad Saood Moazzam Khan, Ahmad Aneeq, Hafiz Muhammad Talha Tahir, Amin Mehmoodi, Jahanzeb Malik, Abida Perveen

**Affiliations:** ^1^ Department of Medicine Ibn e Seena Hospital Kabul Afghanistan

**Keywords:** apolipoprotein A‐I, atherosclerotic cardiovascular disease, cardiovascular risk assessment, high‐density lipoproteins, reverse cholesterol transport

## Abstract

**Background:**

Apolipoprotein A‐I (ApoA‐I), the principal protein component of high‐density lipoproteins (HDL), plays a pivotal role in cardiovascular physiology and has gained increasing attention in atherosclerotic cardiovascular disease (ASCVD). Its involvement extends beyond lipid transport to include anti‐inflammatory and antioxidant mechanisms.

**Objective:**

To comprehensively evaluate the role of ApoA‐I in ASCVD, including its biological functions, clinical relevance as a biomarker, and potential as a therapeutic target.

**Methods:**

A scoping review of the literature was conducted to examine current evidence on ApoA‐I in cardiovascular health and disease. Studies assessing its role in reverse cholesterol transport (RCT), association with cardiovascular outcomes, and emerging therapeutic strategies were included.

**Results:**

ApoA‐I contributes significantly to reverse cholesterol transport and exhibits antioxidant and anti‐inflammatory properties that protect against atherosclerosis. Elevated ApoA‐I levels are consistently associated with reduced risk of major adverse cardiovascular events, supporting its utility as a biomarker for cardiovascular risk assessment. However, variability in HDL particle composition and the influence of confounding factors such as comorbidities and lifestyle limit its interpretability. Therapeutic approaches targeting ApoA‐I, including infusion therapies and mimetic peptides, have shown mixed results in clinical trials, highlighting ongoing challenges.

**Conclusion:**

ApoA‐I remains a promising biomarker and therapeutic target in ASCVD, though its clinical application is complicated by biological and methodological variability. Future research focusing on gene therapies, small molecule modulators, and ApoA‐I mimetics may enhance its functional properties and clinical utility. Integrating ApoA‐I into personalized treatment strategies could improve cardiovascular outcomes and reduce disease burden.

## Graphical abstract

Apolipoprotein A‐I (ApoA‐I), the principal protein of HDL, plays a key role in reverse cholesterol transport, inflammation modulation, and plaque stabilization in atherosclerotic cardiovascular disease. Emerging ApoA‐I–based therapies, including CSL112, enhance cholesterol efflux capacity, though definitive reductions in cardiovascular events remain under investigation in ongoing clinical trials.

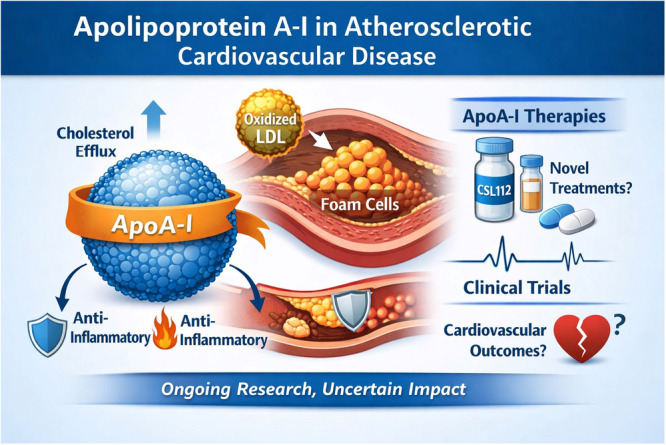

## 1 Introduction

Atherosclerotic cardiovascular disease (ASCVD) remains a leading cause of morbidity and mortality worldwide and is responsible for a substantial proportion of cardiovascular events, including myocardial infarction, stroke, and peripheral artery disease. The condition is characterized by the progressive accumulation of atherosclerotic plaques within arterial walls, resulting in vascular narrowing, inflammation, and impaired blood flow [1, 2]. Plaque rupture can trigger thrombotic events, further worsening clinical outcomes. The global burden of ASCVD continues to increase due to aging populations, unhealthy dietary patterns, sedentary lifestyles, and the rising prevalence of risk factors such as diabetes, hypertension, and dyslipidemia [3, 4].

Lipoproteins play a central role in the pathogenesis of ASCVD by transporting cholesterol and other lipids in the circulation. Low‐density lipoprotein (LDL) contributes to cholesterol deposition within arterial walls and promotes atherosclerotic plaque formation [5, 6]. In contrast, high‐density lipoprotein (HDL) participates in reverse cholesterol transport, a process that removes excess cholesterol from peripheral tissues and returns it to the liver for excretion [7, 8]. While higher HDL levels have traditionally been associated with lower cardiovascular risk, current evidence suggests that HDL functionality may be more important than HDL concentration alone.

Apolipoprotein A‐I (ApoA‐I) is the primary structural and functional protein component of HDL and plays a critical role in mediating its protective effects [9, 10]. ApoA‐I facilitates the formation of nascent HDL particles and promotes cholesterol efflux through interaction with ATP‐binding cassette transporter A1 (ABCA1) on cell membranes. In addition to its role in lipid transport, ApoA‐I exhibits anti‐inflammatory, antioxidant, and antithrombotic properties that may contribute to plaque stabilization and vascular protection [11].

This review aims to examine the role of Apolipoprotein A‐I in cardiovascular outcomes in patients with atherosclerotic cardiovascular disease and to summarize current mechanistic insights and emerging therapeutic perspectives.

## 2 Methods

### 2.1 Study Design

This study was conducted as a scoping review to summarize the available evidence on the role of apolipoprotein A‐I (ApoA‐I) in cardiovascular outcomes related to atherosclerotic cardiovascular disease (ASCVD). The review was guided by established methodological frameworks for scoping reviews, including the approach proposed by Arksey and O'Malley and recommendations from the Joanna Briggs Institute. The objective was to map the existing literature, identify key concepts, and summarize current knowledge regarding ApoA‐I structure, mechanisms, and clinical relevance.

### 2.2 Literature Search Strategy

A comprehensive search of the literature was performed using PubMed/MEDLINE, Scopus, Web of Science, and Google Scholar from database inception to January 2026. The search strategy incorporated combinations of Medical Subject Headings (MeSH) and free‐text terms related to apolipoprotein A‐I and cardiovascular disease, including “Apolipoprotein A‐I,” “ApoA‐I,” “high‐density lipoprotein,” “atherosclerosis,” “cardiovascular disease,” “coronary artery disease,” “myocardial infarction,” “stroke,” and “reverse cholesterol transport.” Boolean operators were applied to refine the search, and the reference lists of relevant reviews and original articles were screened to identify additional studies.

### 2.3 Eligibility Criteria

Studies were considered eligible if they investigated ApoA‐I levels, biological functions, or ApoA‐I–related therapeutic approaches in relation to cardiovascular disease or atherosclerosis. Original research articles, observational studies, clinical trials, systematic reviews, and meta‐analyses published in English were included. Studies that did not address ApoA‐I or cardiovascular outcomes, duplicate publications, conference abstracts without full data, editorials, and case reports were excluded.

### 2.4 Study Selection

All retrieved records were initially screened by title and abstract to determine relevance. Potentially eligible articles were then assessed through full‐text review. Studies were selected based on their relevance to ApoA‐I biology, cardiovascular mechanisms, clinical associations, and therapeutic implications. Any uncertainties regarding eligibility were resolved through discussion to ensure consistency in study selection.

### 2.5 Data Extraction and Synthesis

Key information from the included studies was extracted, including study design, population characteristics, major findings, and reported associations between ApoA‐I and cardiovascular outcomes. The findings were synthesized narratively and organized into thematic sections addressing ApoA‐I structure and function, mechanisms of cardiovascular protection, clinical evidence, and emerging therapeutic strategies. Because the purpose of this scoping review was to map the available evidence, a quantitative meta‐analysis was not performed.

## 3 Discussion

### 3.1 Apolipoprotein A‐I and Cardiovascular Outcomes

Apolipoprotein A‐I (ApoA‐I) has been the focus of numerous clinical investigations because of its central role in HDL metabolism and reverse cholesterol transport [12, 13, 14]. Experimental studies demonstrate that ApoA‐I facilitates cholesterol efflux from macrophages, reduces oxidative stress, and modulates vascular inflammation, all of which are key mechanisms involved in the development and progression of atherosclerosis [15, 16, 17]. These biological properties provide a strong mechanistic basis supporting a potential cardioprotective role for ApoA‐I.

Epidemiological studies have reported an inverse association between ApoA‐I levels and the risk of cardiovascular disease, including coronary artery disease, myocardial infarction, and stroke [18, 19, 20, 21, 22]. ApoA‐I has therefore been proposed as a biomarker that may better reflect HDL functionality compared with HDL cholesterol concentration alone. Several studies have suggested that ApoA‐I levels correlate with cholesterol efflux capacity and plaque stability, indicating its potential value in cardiovascular risk assessment (Tables 1 and 2) [31, 32, 33]. However, much of the existing evidence is observational and may be influenced by confounding clinical factors.

**Table 1:** Main PK/PD and safety fndings of CSL112 Phase 1 studies.

Study characteristics	Intervention	PK Findings	PD Findings	Safety
Easton et al. [23]	Healthy volunteers (*n* = 57); Double‐blind, placebo‐controlled RCT; Single ascending dose (SAD) of CSL112/placebo (5, 15, 40, 70, 105, and 135 mg/kg)	Rapid increase in ApoA‐I with a Tmax of 2 h; biphasic decline; ApoA‐I half‐life (t½) ranged from 39.8 to 99.5 h; sustained ApoA‐I levels above baseline for approximately 3 days	Significant dose‐dependent increases in ApoA‐I concentration; Total cholesterol efflux capacity (CEC) rose by 2.9‐fold; ABCA1‐dependent CEC increased by up to 5.8‐fold; Direct correlation between HDL‐VS area under the effect curve (AUEC0–24) and ApoA‐I area under the curve (AUC0–24)	No adverse events reported
Gille et al. [24]	Healthy volunteers (*n* = 36); Double‐blind, placebo‐controlled RCT; Multiple ascending dose (MAD) of CSL112/placebo (administered once at 3.4 or 6.8 g or twice‐weekly at 3.4 g)	Immediate increase in ApoA‐I and phosphatidylcholine (PC), Tmax at 2 h; sustained ApoA‐I levels above baseline for around 3 days; minimal accumulation observed in the 1‐week infusion, small accumulation in the 2‐week regimen	Dose‐dependent elevations in ApoA‐I; Variable ApoA‐I half‐life; Total CEC increased by 2.6‐fold; ABCA1‐dependent CEC increased by up to 6.3‐fold; Significant correlation between CEC AUEC and ApoA‐I exposure	No signs of major organ toxicity or immunogenicity
Tortorici et al. [25]	Volunteers with normal (*n* = 16) and moderate chronic kidney disease (CKD) (*n* = 16); Matched for age, sex, and weight; Double‐blind, placebo‐controlled RCT; Single infusion of CSL112/placebo (2 or 6 g)	Similar ApoA‐I and PC pharmacokinetic profiles between groups; moderate renal impairment subjects showed slower elimination over 24–48 h	Dose‐dependent increases in ABCA1‐dependent and independent cholesterol efflux, with comparable results across renal groups; Total CEC increased by approximately 2.5‐fold	No significant adverse effects reported
Zheng et al. [26]	Healthy Japanese and Caucasian volunteers (*n* = 34); Double‐blind, placebo‐controlled RCT; Single infusion of CSL112/placebo (Japanese: 2, 4, or 6 g; Caucasian: 6 g)	ApoA‐I levels remained above baseline for 72 h (2 g group) to 144 h (4 and 6 g groups); Dose‐dependent increase in ApoA‐I exposure, comparable profiles between Japanese and Caucasian subjects	Total CEC peaked at 2 h and returned to baseline at varying times depending on dose; ABCA1‐dependent CEC peaked at 2 h, with return to baseline after 8 to 96 h	Two cases of mild hypersensitivity observed (1 in each group)

**Table 2:** Main PK/PD and safety fndings of CSL112 Phase 2 studies.

Study characteristics	Intervention	PK Findings	PD Findings	Safety
Tricoci et al. [27]	Phase 2a, double‐blind, placebo‐controlled RCT; Patients with stable coronary artery disease (CAD) (*n* = 45); Single infusions of CSL112/placebo (1.7, 3.4, 6.8 g)	CSL112 1.7 g: Cmax 0.34 ± 26.9 g/L; AUC0–last 869 ± 8530 mg h/dL; t½ 13.6 ± 81.6 h; CSL112 3.4 g: Cmax 0.77 ± 16.9 g/L; AUC0–last 2040 ± 4100 mg h/dL; t½ 29.6 ± 55.7 h; CSL112 6.8 g: Cmax 1.84 ± 19.1 g/L; AUC0–last 5330 ± 3390 mg h/dL; t½ 49.1 ± 62.1 h	Rapid, dose‐dependent increase in cholesterol efflux capacity (CEC), up to 3.1‐fold compared to placebo; Dose‐dependent increases in total cholesterol (TC) and HDL cholesterol (HDL‐C), peaking at 8 h	No increase in liver enzyme levels; Creatinine levels increased in 75.8% of CSL112 group and 63.6% of placebo group; No viral seroconversion or production of autoantibodies
AEGIS‐I Trial [28]	Phase 2b, double‐blind, placebo‐controlled RCT; Patients with recent myocardial infarction (MI) and normal or mild chronic kidney disease (CKD) (*n* = 1267); Four weekly infusions of CSL112/placebo (2 or 6 g)	CSL112 2 g: 1.29‐fold increase in ApoA‐I; CSL112 6 g: 2.06‐fold increase in ApoA‐I	CSL112 2 g: 1.87‐fold increase in total CEC; 3.67‐fold increase in ABCA1‐dependent CEC; CSL112 6 g: 2.45‐fold increase in total CEC; 4.30‐fold increase in ABCA1‐dependent CEC	Renal impairment in 0.2% of placebo group, 0% in 2 g group, 0.7% in 6 g group; Hepatic impairment in 0% of placebo group, 1.0% in 2 g group, 0.5% in 6 g group; Both CSL112 doses showed non‐inferiority to placebo for renal and hepatic impairment; No significant differences in bleeding, major adverse cardiovascular events (MACE), drug hypersensitivity, or serious adverse events (SAEs)
AEGIS‐I Trial PK/PD Sub‐study [29]	Patients with recent MI and normal or mild CKD (*n* = 63); Four weekly infusions of CSL112/placebo (2 or 6 g)	CSL112 2 g: Biphasic ApoA‐I decline over 48 h; ApoA‐I t½ was 53.9 h after the first infusion and 104 h after the last infusion (mean 53.9 ± 38.7 h); CSL112 6 g: Biphasic ApoA‐I decline over 168 h; PC AUC0–24 mg h/dL was 508 ± 349 (2 g) and 1545 ± 552 (6 g)	CSL112 2 g: twofold increase in total CEC; threefold increase in ABCA1‐dependent CEC; 6.4 mg/dL increase in HDL‐C at 6 h; ABCA1‐dependent CEC returned to baseline after 12 h; ABCA1‐independent CEC returned after 24 h; CSL112 6 g: threefold increase in total CEC; sixfold increase in ABCA1‐dependent CEC; 13.4 mg/dL increase in HDL‐C at 6 h; Both CEC types returned to baseline after 48 h	No major safety concerns reported
CSL112‐2001 Trial [30]	Phase 2, double‐blind, placebo‐controlled RCT; Patients with recent MI ( ≤ 7 days), diabetes mellitus, and moderate CKD (*n* = 83); Four weekly infusions of CSL112/placebo (6 g)	CSL112 6 g: twofold increase in ApoA‐I after the first infusion, similar to subsequent infusions; ApoA‐I levels returned to baseline within 24–48 h; Increase consistent through all infusions	CSL112 6 g: twofold increase in total CEC; Similar increases in ABCA1‐dependent and ABCA1‐independent CEC	No significant adverse events noted

Importantly, previous therapeutic approaches designed to increase HDL cholesterol levels have not consistently translated into reductions in cardiovascular events. Large clinical trials evaluating niacin and cholesteryl ester transfer protein (CETP) inhibitors demonstrated substantial increases in HDL levels but failed to show clear cardiovascular benefit [23, 24, 34]. These findings highlighted the complexity of HDL biology and shifted research attention toward HDL functionality rather than HDL concentration. Within this context, ApoA‐I has gained interest as a potentially more meaningful therapeutic target because of its direct involvement in cholesterol transport and anti‐inflammatory processes [19, 21].

Therapeutic approaches aimed at enhancing ApoA‐I activity, including ApoA‐I mimetic peptides and recombinant HDL formulations, have shown encouraging results in early experimental and clinical studies. These interventions have demonstrated improvements in cholesterol efflux capacity and markers of vascular inflammation [25, 26, 27]. CSL112, a reconstituted ApoA‐I therapy, has been investigated for use in patients following acute myocardial infarction. Early phase trials demonstrated that CSL112 significantly increased cholesterol efflux capacity and was generally well tolerated [28, 29]. These findings support the biological plausibility of ApoA‐I–based therapies during the high‐risk period following acute coronary events.

**Figure 1:**
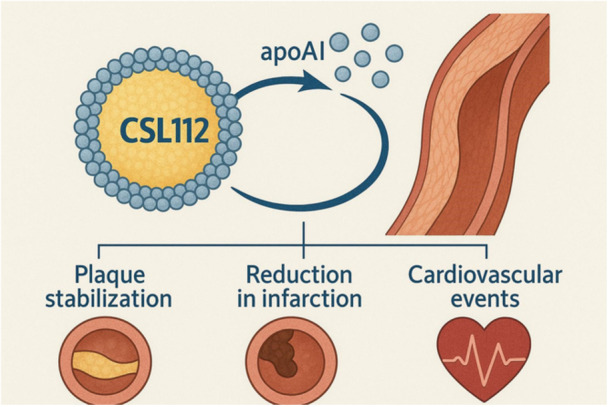
Mechanism of action and potential clinical benefits of CSL112. CSL112, a reconstituted high‐density lipoprotein (HDL) formulation enriched with apolipoprotein A‐I (apoA‐I), promotes cholesterol efflux and vascular repair. This leads to plaque stabilization, reduction in myocardial infarction size, and a decrease in recurrent cardiovascular events.

Nevertheless, improvements in surrogate markers such as cholesterol efflux capacity do not necessarily translate into reductions in major adverse cardiovascular events. The definitive clinical benefit of ApoA‐I‐based therapies remains uncertain, and large outcome trials such as AEGIS‐II are expected to provide more conclusive evidence (Figure 1) [28, 29, 35]. Until such data become available, the therapeutic potential of ApoA‐I should be interpreted cautiously.

Recent research has also highlighted emerging diagnostic indices such as the lectin‐like oxidized low‐density lipoprotein receptor‐1 (LOX‐1), a scavenger receptor that binds oxidized LDL (oxLDL) and promotes its uptake by macrophages, contributing to foam cell formation and atherosclerotic plaque progression; circulating soluble LOX‐1 (sLOX‐1) has therefore been proposed as a potential biomarker for the diagnosis and risk stratification of acute coronary syndromes and other atherosclerotic conditions [36].

### 3.2 Challenges and Limitations in ApoA‐I Research

Despite promising mechanistic insights, several limitations complicate the interpretation of ApoA‐I research. HDL particles are heterogeneous and differ in size, composition, and biological function. Under certain pathological conditions, HDL may become dysfunctional and lose its protective properties [37]. This variability makes it difficult to rely solely on ApoA‐I concentration as an indicator of cardiovascular protection.

In addition, multiple clinical factors can influence ApoA‐I levels and HDL functionality. Metabolic disorders such as diabetes, chronic kidney disease, and systemic inflammation can alter HDL structure and impair cholesterol efflux capacity [38]. Genetic variation, lifestyle factors, and medication use may also affect ApoA‐I levels, potentially confounding associations observed in observational studies [39]. These issues highlight the need for more comprehensive approaches that evaluate HDL functionality alongside ApoA‐I measurements.

Therapeutic development targeting ApoA‐I also faces several challenges. While early studies of ApoA‐I mimetics and recombinant HDL have shown improvements in surrogate biomarkers, the translation of these findings into meaningful clinical outcomes remains uncertain. Previous experiences with HDL‐raising therapies emphasize the importance of large randomized trials evaluating hard cardiovascular endpoints rather than relying solely on biomarker changes [23, 24, 34].

### 3.3 Future Directions

Future research on ApoA‐I is increasingly focused on improving HDL functionality and developing therapies that enhance cholesterol efflux and vascular protection. Novel approaches include gene‐based therapies, ApoA‐I mimetic peptides, and small‐molecule modulators designed to improve ApoA‐I expression or activity. These strategies aim to strengthen the biological processes involved in reverse cholesterol transport and plaque stabilization.

Another promising area involves the integration of ApoA‐I into precision medicine approaches for cardiovascular risk assessment. Because ApoA‐I reflects aspects of HDL functionality, its measurement may help identify individuals at higher risk who might benefit from targeted therapies. Combining ApoA‐I with other biomarkers and clinical parameters could improve risk prediction models and guide individualized treatment strategies.

Ongoing large‐scale clinical trials will be crucial in determining whether ApoA‐I‐based interventions can meaningfully reduce cardiovascular events. Studies such as AEGIS‐II are expected to provide important evidence regarding the clinical impact of therapies designed to enhance cholesterol efflux during the early period following acute coronary syndromes (Figure 1) [28, 29, 35]. Continued investigation into HDL biology, ApoA‐I functionality, and patient selection will be essential for translating mechanistic insights into effective cardiovascular therapies.

## 4 Conclusion

Apolipoprotein A‐I plays a fundamental role in HDL metabolism and contributes to multiple protective mechanisms involved in atherosclerosis, including reverse cholesterol transport, antioxidant activity, and modulation of vascular inflammation. Observational and mechanistic studies suggest that ApoA‐I is associated with favorable cardiovascular profiles and may serve as a useful biomarker reflecting HDL functionality and cardiovascular risk (Tables 1 and 2) [18, 19, 20, 21, 22]. Emerging therapies targeting ApoA‐I have demonstrated encouraging effects on surrogate markers such as cholesterol efflux capacity [25, 26, 27, 28, 29]. However, definitive evidence demonstrating reductions in major cardiovascular events remains limited. Experiences with previous HDL‐targeted therapies highlight the complexity of translating biological insights into effective clinical treatments [23, 24, 34]. Ongoing randomized trials will be essential to determine whether ApoA‐I–based therapies can provide meaningful cardiovascular benefit in patients with ASCVD.

## Author Contributions

**Awais Habib:** Supervision, methodology, writing. **Muhammad Saood Moazzam Khan:** Project administration, writing, revision. **Ahmad Aneeq:** Investigation, software, resources, revision. **Hafiz Muhammad Talha Tahir:** Writing, methodology. **Amin Mehmoodi:** Software, supervision. **Jahanzeb Malik:** Supervision, writing, revision, methodology. **Abida Perveen:** Supervision, writing, revision, methodology, software.

## Ethics Statement

The authors have nothing to report.

## Funding

The authors have nothing to report.

## Conflicts of Interest

The authors declare no conflicts of interest.

## Data Availability

Data sharing not applicable to this article as no datasets were generated or analysed during the current study.
